# Dietary Iron Overload Abrogates Chemically-Induced Liver Cirrhosis in Rats

**DOI:** 10.3390/nu10101400

**Published:** 2018-10-02

**Authors:** Machi Atarashi, Takeshi Izawa, Mutsuki Mori, Yohei Inai, Mitsuru Kuwamura, Jyoji Yamate

**Affiliations:** Laboratory of Veterinary Pathology, Osaka Prefecture University, 1-58 Rinku Orai Kita, Osaka 598-8531, Japan; blue.ocean-orca-@live.jp (M.A.); sxc01040@edu.osakafu-u.ac.jp (M.M.); yohei.inai.0123@gmail.com (Y.I.); kuwamura@vet.osakafu-u.ac.jp (M.K.); yamate@vet.osakafu-u.ac.jp (J.Y.)

**Keywords:** apoptosis, dietary iron overload, liver cirrhosis, thioacetamide

## Abstract

Chronic liver disease is an intractable disease, which can progress to cirrhosis and hepatocellular carcinoma. Hepatic iron overload is considered to be involved in the progression of chronic liver diseases; however, the mechanism remains to be elucidated. Here we investigate the role of dietary iron overload using chemically-induced liver cirrhosis model. Rats were fed a high-iron or standard diet and were injected intraperitoneally with thioacetamide (TAA) or saline twice a week for 20 weeks. Rats with TAA treatment (TAA group) had progressive liver cirrhosis characterized by persistent hepatocellular injury, mononuclear cell inflammation and bridging fibrosis; these lesions were markedly reduced in rats with iron feeding and TAA treatment (Fe-TAA group). Rats with iron feeding alone (Fe group) had no evidence of liver injury. Hepatic expression of cleaved caspase-3, but not phospho-RIP3, was decreased in Fe-TAA group compared with that in TAA group. The number of TUNEL-positive (terminal deoxynucleotidyl transferase dUTP nick end labeling) apoptotic hepatocytes was lower in the Fe-TAA group than in the TAA group. Hepatic xenobiotic metabolism and lipid peroxidation were shown to be less related to the abrogation of liver cirrhosis. Our results suggested that dietary hepatic iron overload abrogates chemically-induced liver cirrhosis in rats, which could partly involve decreased hepatocellular apoptosis.

## 1. Introduction

Cirrhosis is an intractable, advanced liver disease resulting from chronic liver diseases (CLD) such as viral hepatitis, alcoholic hepatitis and non-alcoholic steatohepatitis. It can progress to hepatocellular carcinoma (HCC), the fifth most common tumor and the second most cause of cancer-related death in the world [1]. The prevalence of cirrhosis among patients with HCC is estimated to be 85–95%. Global liver cirrhosis deaths were more than one million in 2010 [2]. Liver cirrhosis is thus a significant global health burden.

Iron is an essential micronutrient for all living organisms. It has important roles in oxygen transport, oxidative phosphorylation, and other enzymatic functions [3,4]. However, once iron exceeds the storage capacity of the body, excess iron can accumulate in the liver, heart and endocrine glands, leading to damage and/or dysfunction to these vital organs [5]. In physiological condition, hepatocytes have a high capacity for iron storage [6,7]. Hepatocytes produce hepcidin, the master regulator of systemic iron homeostasis. Hepcidin negatively regulates iron efflux via internalization and subsequent degradation of the iron exporter ferroportin, located on the basolateral membrane of duodenal enterocyte, macrophage and hepatocyte. Repeated and/or persistent liver damage can perturb and decrease hepcidin production, resulting in secondary (acquired) iron overload [3,4,8]. Hepatic iron overload can lead to further liver damage via the production of reactive oxygen species (ROS). Thus, iron overload secondary to CLD is regarded as an important risk factor for progression of liver disease [8,9].

Hepatic iron overload is reported to occur in more than one third of CLD patients including chronic viral hepatitis and non-alcoholic fatty liver disease (NAFLD) [10,11,12]. Liver iron deposition is considered as a predictor of progression and clinical outcomes in advanced chronic hepatitis C [13]. Presence of stainable iron in hepatic non-parenchymal (reticuloendothelial) cells is associated with the presence of non-alcoholic steatohepatitis in patients with NAFLD [11,14]. In addition to these acquired conditions, hepatic iron overload occurs primarily in hereditary hemochromatosis, a genetic disorder caused by mutations in genes of the hepcidin–ferroportin axis [5]. This disorder is characterized by excess hepatic iron accumulation with liver damage, and progression of disease to cirrhosis and hepatocellular carcinoma.

However, the mechanism by which hepatic iron overload leads to the progression of CLDs remains to be elucidated. Therefore, here we investigate the pathophysiological role of iron overload in a rat model of chemically-induced liver cirrhosis. Thioacetamide (TAA) is a well-known hepatotoxicant that can induce centrilobular necrosis by a single injection and liver fibrosis/cirrhosis by long-term repeated injections [15,16]. Liver pathology of this rat cirrhosis model has been well characterized by our previous studies [16,17,18,19,20].

## 2. Materials and Methods

### 2.1. Animals

Six-week-old male F344/DuCrlCrlj rats (Charles River Laboratories Japan, Yokohama, Japan) were divided into control (cont.), TAA, TAA with high iron (Fe-TAA) and high iron (Fe) groups. Rats in cont. and TAA groups were fed a standard diet (DC-8, containing 0.02% iron; CLEA Japan, Tokyo, Japan), while rats in Fe-TAA and Fe groups were fed a high-iron diet (containing 1% iron; Oriental Yeast Co., Ltd., Tokyo, Japan). Concentration of dietary iron was determined based on the results of our pilot study. Supplement of 1% iron in the diet can induce increased serum iron with nearly saturated transferrin binding and increased liver iron content without hepatocellular damage; these phenotypes are required for the present study. Food and water were provided ad libitum. Rats were given an intraperitoneal injection of TAA (100 mg/kg body weight) or saline twice a week. Rats were deeply anesthetized by isoflurane, and the whole blood and liver were sampled at weeks 6 (early fibrosis stage) and 20 (advanced cirrhosis stage) after the initial injection. All animal procedures were approved by the Animal Care and Use Committee at Osaka Prefecture University (code no. 23–57) and were performed according to the institutional guidelines.

### 2.2. Biochemical Analyses

Blood samples collected from the abdominal aorta were separated by centrifugation (3000 rpm, 5 min), and serum was isolated for analyses. Clinical chemistry and liver iron content were analyzed in SRL Inc. (Tokyo, Japan) as previously reported [15,21]. Malondialdehyde content in the liver was measured by thiobarbituric acid reacting substances (TBARS) method (Nikken SEIL Co., Ltd., Shizuoka, Japan).

### 2.3. Histopathology

The left lateral lobe of the liver was fixed in 10% neutral-buffered formalin, routinely processed, embedded in paraffin, cut at 5 µm and stained with hematoxylin and eosin (HE) for histopathological examination, and with Berlin blue and Sirius red for detection of iron and collagen deposition, respectively.

### 2.4. Immunohistochemistry

Antibodies against CD3 (clone F7.2.38; DAKO, Glostrup, Denmark) for T cells and CD68 (clone ED1; Bio-Rad, Hercules, CA, USA) for macrophages were used for immunohistochemistry. Deparaffinized sections were pretreated with microwave in Tris-EDTA buffer (pH 9.0) and with proteinase K (DAKO) (100 μg/mL, 37 °C, 10 min) for CD3 and CD68, respectively. Sections were then incubated with 5% skim milk in phosphate buffered saline (PBS) for 15 min and with primary antibody for 1 h, followed by 1 h incubation with peroxidase-conjugated secondary antibody (Histofine simple stain MAX PO; Nichirei Biosciences, Tokyo, Japan). Positive reactions were visualized with 3,3′-diaminobenzidine (DAB; Nichirei Biosciences; Tokyo, Japan). Sections were lightly counterstained with hematoxylin. In order to evaluate hepatocellular apoptosis, liver sections were subjected to terminal deoxynucleotidyl transferase dUTP nick end labeling (TUNEL) method as previously described [19,20]. The number of TUNEL-positive apoptotic hepatocytes was counted; more than 500 hepatocytes were analyzed in each animal to obtain reliably quantitative data.

### 2.5. Western Blot

Liver samples were homogenized in RIPA buffer (20 mM Tris-HCl, pH 7.5, 150 mM NaCl, 1 mM EDTA, 1 mM EGTA, 1% NP-40, 0.1% deoxycholate, 0.1% SDS, 1 mM NaF, 100 μM Na_3_VO_4_, 1 mM phenylmethylsulfonyl fluoride, and proteinase inhibitor cocktail; Nakarai Tesque, Kyoto, Japan). After centrifugation at 13,000× *g* for 10 min, the supernatant was mixed with an equal volume of 2× SDS sample buffer (Cosmo Bio, Tokyo, Japan) containing 10% 2-mercaptoethanol (Bio-Rad), and then boiled for 5 min. Samples were separated on gradient (5–20%) or 10% polyacrylamide gels and transferred to polyvinylidene difluoride membranes (Bio-Rad). The membranes were blocked by 5% skim milk in PBS and reacted with anti-cytochrome P450 2E1 (CYP2E1) (1:1000; #Hpa009128, Sigma-Aldrich Co., St. Lois, MN, USA), anti-flavin containing monooxygenase (FMO)-3 (1:2000; #EPR6968, Abcam, Cambridge, UK), anti-cleaved caspase-3 (1:1000; #9664, Cell Signaling Technology, Danvers, MA, USA), anti-phosphorylated receptor-interacting protein 3 (p-RIP3) (1:1000; #ab195117, Abcam, Cambridge, UK), anti-B-cell lymphoma protein 2-associated X (Bax) (1:500; #14796, Cell Signaling Technology), anti-α-tubulin (1:1000; #2125, Cell Signaling Technology) and anti-porin/voltage-dependent anion-selective channel protein 1 (VDAC1) (1:1000; #ab15895, Abcam) antibodies overnight at 4 °C. The membranes were then incubated with peroxidase-conjugated secondary antibody (Histofine simple stain MAX PO; Nichirei Biosciences, Tokyo, Japan) for 30 min and were reacted with ECL Prime Western Blotting Detection Reagent (GE Healthcare, Chicago, IL, USA). Signals were detected with LAS-4000 imaging system (GE Healthcare).

### 2.6. Statistical Analysis

Data are presented as mean ± standard deviation (SD). Statistical analyses were performed using Prism software (version 7.0c; Graphpad, CA, USA) with Tukey’s or Sidak’s multiple comparison. Pearson’s correlation coefficient was used for correlation analysis. A value of *p* < 0.05 was considered statistically significant.

## 3. Results

### 3.1. Dietary Iron Overload Abrogates the Pathological Phenotype of Chemically Induced Liver Cirrhosis

#### 3.1.1. Biochemical Findings

**Figure 1:**
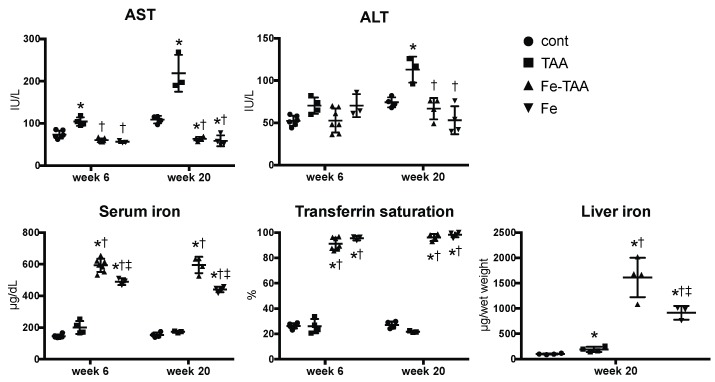
Biochemical data of serum aminotransferase (ALT), aspartate aminotransferase (AST), iron, transferrin saturation at weeks 6 and 20 and liver iron content at week 20. Data are expressed as mean ± standard deviation (SD). * *p* < 0.05 vs. control; † vs. thioacetamide (TAA); ‡ vs. Fe-TAA at the same time point, by Sidak’s multiple comparison.

Clinical chemistry showed a significant increase in serum alanine aminotransferase (ALT) and aspartate aminotransferase (AST) in the TAA group compared to those in cont. and Fe groups at week 20 (Figure 1); the levels of AST and ALT were significantly lower in the Fe-TAA group than in the TAA group at week 20. AST levels also increased in the TAA group at week 6, compared to those in the other three groups. Serum iron, transferrin saturation and liver iron increased significantly in the Fe-TAA and Fe groups compared to the control and the TAA groups. Liver iron content was also increased by TAA administration; it was higher in the TAA and Fe-TAA groups than the control and Fe groups, respectively. Likewise, serum iron levels were higher in the Fe-TAA group than that in the Fe group.

#### 3.1.2. Pathological Findings

**Figure 2:**
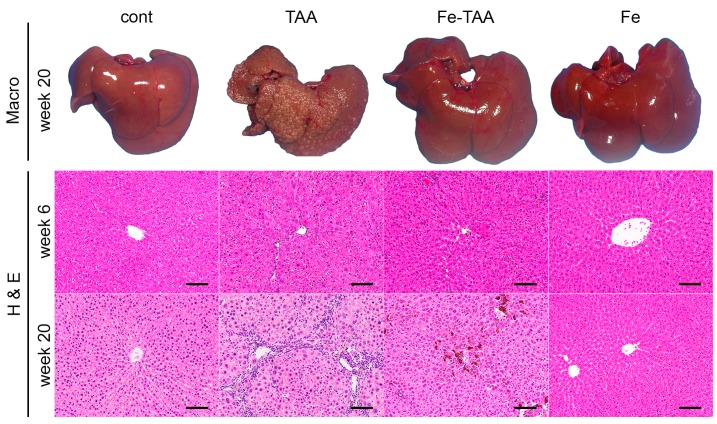
Macroscopic (**upper**) and hematoxylin and eosin (H & E; **middle** and **lower**) images of the liver at weeks 6 and 20. Bar: 100 µm.

**Figure 3:**
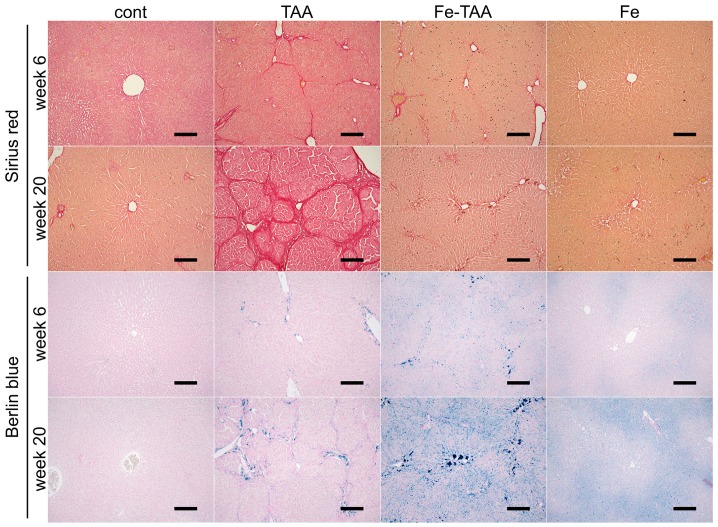
Sirius red (**upper**) and Berlin blue (**lower**) images of the liver at weeks 6 and 20. Collagen deposits stain red with Sirius red, while iron deposits stain blue with Berlin blue. Bar: 200 μm.

Grossly, the liver in the TAA group at week 20 had a discoloration and multinodular appearance, indicating advanced liver cirrhosis (Figure 2, upper); these findings were absent in Fe-TAA group. The liver in the Fe-TAA and Fe groups was brownish in color, reflecting the increased iron content. These gross abnormalities were subtle at week 6. Histopathologically, there were hepatocellular karyocytomegaly and mild bridging fibrosis in the liver of the TAA group at week 6 (Figure 2, lower), which was less prominent in the Fe-TAA group. The fibrosis was more clearly visualized by Sirius red stain (Figure 3, upper). At week 20, the liver in the TAA group had extensive bridging fibrosis with formation of pseudolobules (Figure 2 and Figure 3), consistent with the gross appearance of advanced liver cirrhosis. Marked hepatocellular karyocytomegaly and oval cell hyperplasia were also observed in the TAA group. The bridging fibrosis was much less prominent in the Fe-TAA group than in the TAA group (Figure 2 and Figure 3). There were aggregates of macrophages laden with brown pigment (hemosiderin) in the sinusoid of the Fe-TAA group. No marked abnormality was observed in the liver of the Fe group at either time point.

Iron deposition was undetectable with Berlin blue stain in control liver (Figure 3, lower). Iron deposition was intense in sinusoidal cells (mainly Kupffer cells/macrophages) and mild to moderate in hepatocytes in the liver of Fe group at weeks 6 and 20; within the hepatic lobule it was more intense in the periportal area (zone 1) than in the centrilobular area (zone 3). In the TAA group, iron deposits were observed mainly in macrophages in the fibrotic lesions, which was more pronounced in the Fe-TAA group. Hepatocellular iron staining was more intense in the Fe-TAA group than in Fe group. These findings were consistent with the biochemical liver iron content described above (Figure 1).

**Figure 4:**
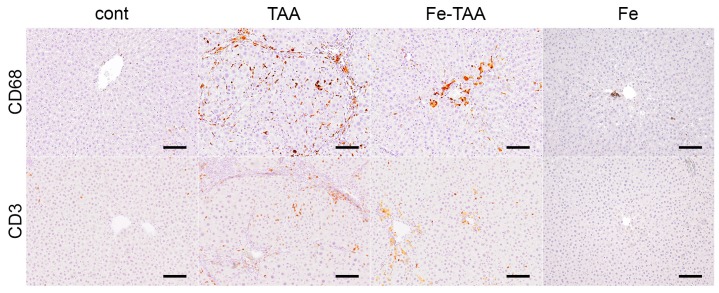
Immunohistochemistry for CD68 (**upper**) and CD3 (**lower**) in the liver at week 20. Note hemosiderin deposits (yellow-brown pigment, apart from immunohistochemical signals) in the Fe-TAA group. Bar: 100 μm.

We next analyzed the degree of inflammatory cell infiltration within the hepatic lesions at week 20, by immunohistochemistry for CD3 (T lymphocytes) and CD68 (macrophages/Kupffer cells). There were a small number of CD3-positive T cells and CD68-positive macrophages/Kupffer cells in the liver of the control and Fe groups (Figure 4). A large number of CD3-positive T cells and CD68-positive macrophages were observed in the TAA group, which was markedly reduced in the Fe-TAA group. The degree of inflammation was consistent with that of serum transaminase levels and liver histopathology.

### 3.2. The Abrogation of Liver Cirrhosis by Dietary Iron Overload Is More Related to Changes in Apoptosis Than in Xenobiotic Metabolism and Oxidative Stress

#### 3.2.1. Changes in Thioacetamide (TAA)-Metabolizing Enzymes 

**Figure 5:**
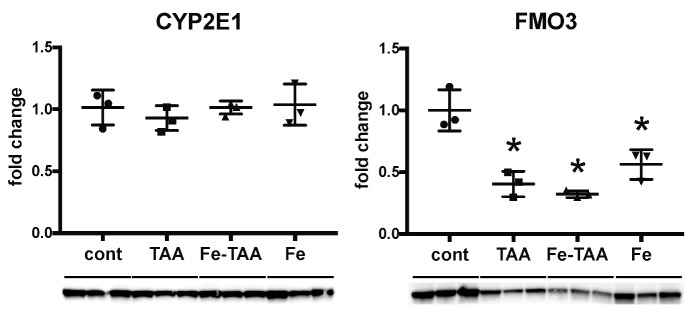
Hepatic expression of anti-cytochrome P450 2E1 (CYP2E1) and anti-flavin containing monooxygenase (FMO-3) at week 6. Data are expressed as mean ± SD. * *p* < 0.05 vs. cont., by Tukey’s multiple comparison.

TAA is metabolized in the liver by CYPs (particularly CYP2E1) and FMOs, leading to production of toxic metabolites thioacetamide S-oxide and thioacetamide S,S-dioxide [22,23]. FMO3 is abundant and the major FMO in the rat liver [24,25]. We thus analyzed hepatic expression of CYP2E1 and FMO3 in the early stage of chronic liver injury at week 6, in order to understand the mechanism by which the TAA-induced liver lesions were reduced by high-iron diet feeding. Expression of CYP2E1 did not change between the four groups (Figure 5). Expression of FMO3 decreased in the TAA, Fe-TAA and Fe groups compared to the control group, with no significant difference between the TAA and Fe-TAA groups. These results suggest no direct link between the changes in metabolic enzyme expression and liver pathology.

#### 3.2.2. Changes in Oxidative Stress

**Figure 6:**
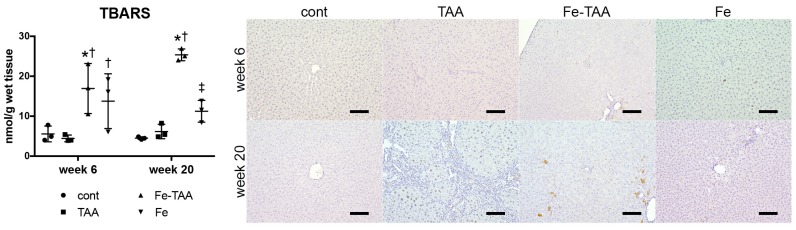
Hepatic thiobarbituric acid reacting substances (TBARS) levels (left) at weeks 6 and 20. Data are expressed as mean ± SD. * *p* < 0.05 vs. cont.; † vs. TAA; ‡ vs. Fe-TAA at the same time point, by Sidak’s multiple comparison. Immunohistochemistry for γH2A.X (right) in the liver at weeks 6 and 20. Note hemosiderin deposits (yellow-brown pigment, apart from immunohistochemical signals) in Fe-TAA group. Bar: 100 µm.

We next investigated hepatic TBARS levels (a marker for lipid peroxidation) and immunoreactivity for phospho-histone H2A.X (γH2A.X; a marker for DNA damage). Hepatic TBARS levels increased in the Fe-TAA group compared to those in the control and TAA groups at weeks 6 and 20 (Figure 6, left). TBARS levels in the Fe group were lower than those in the Fe-TAA group at week 20. Immunohistochemistry revealed that there were few γH2A.X-positive cells in the liver of the control, Fe-TAA and Fe groups while there were some γH2A.X-positive hepatocytes in the TAA group at week 20 (Figure 6, right). These data suggest that oxidative stress does not contribute directly to the attenuation of TAA-induced liver lesions in the Fe-TAA group.

#### 3.2.3. Changes in Cell Death

**Figure 7:**
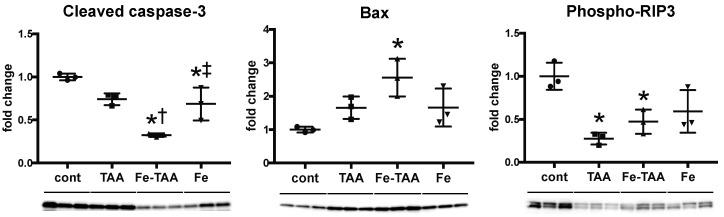
Western blot data for cleaved caspase-3 (apoptosis marker), Bax (mitochondrial pro-apoptotic protein) and phospho-RIP3 (necroptosis marker) using liver samples at week 6. Data are normalized by α-tubulin for cleaved caspase-3 and phospho-RIP3 and by porin (anti-porin/voltage-dependent anion-selective channel protein 1, VDAC1) for Bax, and expressed as mean ± SD. * *p* < 0.05 vs. cont.; † vs. TAA; ‡ vs. Fe-TAA, by Tukey’s multiple comparison.

**Figure 8:**
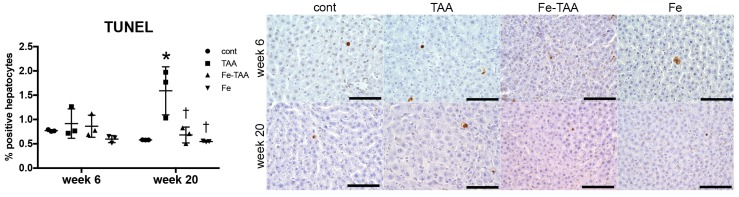
The number of TUNEL-positive (terminal deoxynucleotidyl transferase dUTP nick end labeling) hepatocytes (left) at weeks 6 and 20. * *p* < 0.05 vs. cont.; † vs. TAA at the same time point, by Sidak’s multiple comparison. Representative images of TUNEL method (right) in the liver at weeks 6 and 20. Bar: 100 µm.

We finally investigated hepatic expression of cell death markers at week 6. Expression of cleaved caspase-3, a central molecule for caspase-dependent apoptosis [26], decreased in the Fe-TAA and Fe groups compared to cont. groups (Figure 7); it was lower in the Fe-TAA group than in the TAA and Fe groups. Bax is a pro-apoptotic protein that can form pores in the mitochondrial outer membrane, leading to the release of cytochrome c, the initial step for apoptosis [27]. Bax expression increased in the Fe-TAA group compared to the control group, inconsistent with the decreased caspase-3 cleavage in this group. Expression of phospho-RIP3, a marker for necroptosis [28], decreased in the TAA and Fe-TAA groups compared to the cont. group, with no significant difference between the TAA and Fe-TAA groups. The number of TUNEL-positive apoptotic hepatocytes increased in TAA group compared with that in cont. group at week 20, which was completely suppressed in Fe-TAA group (Figure 8). The number of TUNEL-positive apoptotic hepatocytes was positively correlated with the level of serum AST (*p =* 0.0024, *r* = 0.6015) but not ALT (*p =* 0.1662, *r* = 0.2987). There was no significant change in TUNEL-positive hepatocytes between the groups at week 6. These data raise the possibility that the decreased hepatocellular apoptosis can be related with the attenuation of TAA-induced liver lesions in Fe-TAA group, and that regulation of apoptotic pathway is partly altered in the Fe-TAA group.

## 4. Discussion

In this study we showed that development of TAA-induced liver cirrhosis, characterized by persistent hepatocellular injury, inflammation and bridging fibrosis, is abrogated by feeding of high-iron diet in rats. The rats in the Fe-TAA group had a marked reduction in these liver lesions, despite the presence of systemic and hepatic iron overload. Our results are quite different from the commonly accepted notion that hepatic iron overload can worsen and accelerate CLD such as chronic viral hepatitis C [13]. Our results are also opposite to the results of a mouse study showing that carbon tetrachloride-induced liver fibrosis is enhanced by dietary hepatic iron overload with supplementation of carbonyl iron [29].

It is well known that excess iron can induce oxidative tissue injury in the liver. When body iron increases more than the buffering capacity of serum transferrin, non-transferrin-bound iron appears [4,30]. The highly reactive forms of iron (labile plasma iron) is incorporated by hepatocytes and then accumulate in their cytoplasm; the labile cellular iron fuels generation of ROS via Fenton chemistry, leading to consequent damage to DNA, proteins and membranes and eventually to cell death. Interestingly, our results showed a decreased hepatocellular apoptosis in the liver of the Fe-TAA group compared with that in the TAA group, despite the presence of hepatic iron overload with increased lipid peroxidation. While many studies have focused on the mechanisms of iron-induced cell death such as apoptosis, necroptosis and ferroptosis [31,32], the anti-apoptotic role of iron is largely unknown. A few studies suggest that preconditioning of sublethal dose of iron can prevent apoptosis against multiple stresses in vitro and in vivo [33,34,35,36]. In rodent models of dietary hemochromatosis, animals with a liver iron content of >2000 μg/g (wet weight) have a marked elevation of serum transaminases while those with a liver iron content of <1500 μg/g have no transaminase elevation [37,38,39]. Since the liver of the Fe group had an iron content of 916 ± 135 μg/g and did not have any evidence of hepatocellular injury, the dietary iron overload itself is considered to be non-toxic to the liver in this study. TAA-induced liver injury is shown to be initiated by sporadic hepatocellular apoptosis, followed by extensive centrilobular necrosis with inflammatory reactions [40]. It is possible that the dietary iron overload could prevent TAA-induced hepatocellular apoptosis, thereby leading to the subsequent reduction of necrosis, inflammation and fibrosis. Further mechanistic study (e.g., hepatocyte culture with treatment of sublethal iron and multiple hepatotoxic insults) is needed to determine the molecular mechanism for the protection of liver injury by iron overload.

In the present study we investigated the possibility of the involvement of metabolism enzymes in the abrogation of TAA-induced liver cirrhosis. Metabolic enzymes such as CYP2E1 and FMOs play an important role in the development of TAA-induced hepatotoxicity. CYP2E1-null mice have no evidence of liver injury after an acute hepatotoxic dose of TAA [22]. Treatment with methimazole, a competitive FMO inhibitor, suppresses hepatocellular injury and proinflammatory cytokine production in rats [41]. However, our data showed no significant difference in the expression of CYP2E1 or FMO3 between the Fe-TAA and TAA groups. As the protein expression and enzyme activity of CYP2E1 are shown to be changed correspondingly in rats [42,43], our results suggest that the TAA-metabolizing enzymes are less likely to be involved in the abrogation of liver disease by dietary iron overload in this model.

Given the finding that iron deposits are more intense in sinusoidal non-parenchymal cells than in parenchymal hepatocytes in rats fed a high-iron diet, it is possible that not only hepatocytes but also non-parenchymal cells such as Kupffer cells and hepatic stellate cells (HSCs) can participate in the abrogation of liver cirrhosis by dietary iron overload. We previously showed that dietary iron supplementation enhances steatohepatitis in a rat model of nonalcoholic steatohepatitis, associated with overproduction of proinflammatory cytokine presumably by iron-laden activated Kupffer cells/macrophages [21]. However, in the present study the activation of Kupffer cells/macrophages is less prominent in rats in the Fe-TAA group than in the TAA group.

Cytoglobin is the fourth mammalian globin following hemoglobin, myoglobin and neuroglobin, originally found in rat HSCs [44]. Cytoglobin has heme iron in its structure and can bind oxygen and nitric oxide [45]. Its expression is specific in HSCs in the liver and increases with progression of TAA-induced liver fibrosis [44]. Cytoglobin has peroxidase activity catabolizing hydrogen peroxide and lipid peroxides, suggesting a possible role as a ROS scavenger. A study using recombinant adeno-associated virus vector demonstrated that overexpression of cytoglobin prevents free radical-induced HSC activation and liver fibrosis in rats [46]. However, in the present study, hepatic lipid peroxidation was increased in the Fe-TAA group compared to that in the TAA group, despite the marked suppression of liver fibrosis, suggesting that cytoglobin may be less involved in the suppression of liver fibrosis in this model.

It is important to determine whether the dietary iron overload can also abrogate liver injury induced by other chemicals/treatments. The study using multiple hepatotoxic compounds (e.g., carbon tetrachloride, acetaminophen) is in progress to elucidate the pathological roles of dietary iron overload in chemically-induced liver disease.

## 5. Conclusions

This study suggested that dietary iron overload abrogates rat liver cirrhosis induced by repeated injections of TAA, characterized by a significant reduction of hepatocellular injury, inflammation and liver fibrosis. Decreased hepatocellular apoptosis could be partly related to the abrogation of the liver disease.

## Abbreviations

The following abbreviations are used in this manuscript:

CLD	chronic liver disease
HCC	hepatocellular carcinoma
ROS	reactive oxygen species
NAFLD	non-alcoholic fatty liver disease
TAA	thioacetamide
TBARS	thiobarbituric acid reacting substances
PBS	phosphate buffered saline
TUNEL	terminal deoxynucleotidyl transferase dUTP nick end labeling
CYP	cytochrome P450
FMO	flavin containing monooxygenase
p-RIP3	phosphorylated receptor interacting protein 3
Bax	B-cell lymphoma protein 2-associated X
VDAC1	voltage-dependent anion-selective channel protein 1
ALT	alanine aminotransferase
AST	aspartate aminotransferase
HSC	hepatic stellate cell
